# Validating the Use of Google Trends to Enhance Pertussis Surveillance in California

**DOI:** 10.1371/currents.outbreaks.7119696b3e7523faa4543faac87c56c2

**Published:** 2015-10-19

**Authors:** Simon Pollett, Nicholas Wood, W. John Boscardin, Henrik Bengtsson, Sandra Schwarcz, Kathleen Harriman, Kathleen Winter, George Rutherford

**Affiliations:** Marie Bashir Institute for Infectious Diseases and Biosecurity, University of Sydney, NSW, Australia; Department of Epidemiology & Biostatistics, University of California at San Francisco, California, USA; National Centre for Immunisation Research and Surveillance of Vaccine Preventable Diseases, The Children's Hospital at Westmead, Sydney, NSW, Australia; Discipline of Paediatrics and Child Health, University of Sydney, NSW, Australia; Department of Epidemiology & Biostatistics, University of California at San Francisco, California, USA; Department of Medicine, University of California at San Francisco, California, USA; Department of Epidemiology & Biostatistics, University of California at San Francisco, California, USA; Department of Epidemiology & Biostatistics, University of California at San Francisco, California, USA; California Department of Public Health, Richmond, California, USA; California Department of Public Health, Richmond, California, USA; Department of Epidemiology & Biostatistics, University of California at San Francisco, California, USA

## Abstract

Introduction and Methods: Pertussis has recently re-emerged in the United States. Timely surveillance is vital to estimate the burden of this disease accurately and to guide public health response. However, the surveillance of pertussis is limited by delays in reporting, consolidation and dissemination of data to relevant stakeholders. We fit and assessed a real-time predictive Google model for pertussis in California using weekly incidence data from 2009-2014.

Results and Discussion: The linear model was moderately accurate (*r *= 0.88). Our findings cautiously offer a complementary, real-time signal to enhance pertussis surveillance in California and help to further define the limitations and potential of Google-based epidemic prediction in the rapidly evolving field of digital disease detection.

## Background

Pertussis has recently re-emerged in the United States, with state incidence as high as 65.3 per 100,000 persons in 2013 and over 48,000 cases reported in 2012^1^. The Centers for Disease Control and Prevention (CDC) recently described pertussis as “the most poorly controlled vaccine-preventable bacterial disease in the US”^2^ and a number of pertussis-related infant deaths have been reported^3^^,^ 4.Timely surveillance is vital to estimate the burden of this disease accurately and to guide public health response. However, the surveillance of pertussis is limited by delays in reporting, consolidation and dissemination of data to relevant stakeholders.

Recent years have seen the assessment of Google search query trends as a real-time surveillance method for diseases such as influenza^5^ and dengue^6^, although they have never been validated in actual public health practice and significant errors exist with the predictive accuracy of Google Flu Trends^7^^,^ 8 and Google Dengue Trends models^6^^,^ 9.

We sought to fit and assess the accuracy of a pertussis Google predictive model in California, a state that has experienced two recent large epidemics of pertussis^10^.

## Methods

We obtained weekly pertussis case counts in California from August 2009 through July 2014 from the California Department of Public Health, using standard case definitions^10^^,^ 11. Population denominators were obtained from the Californian Department of Finance^12^, with intercensal weekly adjustment. We downloaded weekly normalized pertussis-related Google search term trends by Google users in California from the Google Trends website. Google normalizes all search term trend data by expressing search frequencies as the number of searches about a certain term in an epidemiological week over all searches about any term in that same week^13^. The Google Correlate webtool was used to identify a maximum number of terms regarding pertussis epidemiology, symptoms, diagnosis, treatment and prevention, including misspelled and lay terms^13^^,^ 14. Google only provides lower-volume search terms to a monthly (rather than weekly) resolution and in these cases each epidemiology week falling entirely into that month (or with four or more days falling into that month) was assigned the monthly normalized search volume.

Adopting a methodology used for the Google Dengue and Google Flu Trend models^5^^,^ 6, we divided the incidence trend dataset into a training dataset for January 2009 - December 2012 (containing a pertussis epidemic wave) and a hold-out dataset for January 2013 - July 2014 (containing a subsequent major epidemic wave). We ranked candidate Google predictors based on Pearson correlation with the Californian pertussis incidence trends during the training period. We fit a linear regression model with candidate Google search predictors added sequentially (in order of descending rank) to the model and retained if they improved the model adjusted R^2^ (the coefficient of determination, that is, the proportion of variance explained by the model, with a penalty adjustment by the number of predictors in the model) which allows for a more conservative estimate of prediction error for linear regression models^15^.

Due to the risk of over-fitting and because the adjusted R^2^ may still underestimate prediction error, we also required that predictors improved model fit based on a ten-fold internal cross-validation, an approach described in full biostatistical detail elsewhere^15^. Briefly, the training dataset was randomly divided into ten mutually exclusive subsets. Each of the ten subsets was withheld in turn and the model was estimated using the remaining nine subsets of data, thus generating one-tenth of the model fitted outcomes at a time. The complete set of fitted values from this process were compared to the original full training set of data by Pearson correlation. This process was repeated ten times to get a mean Pearson correlation value. When a term did not satisfy these criteria for inclusion (that is, improvement of the linear model adjusted R^2^ and improvement of the *r* derived from the ten-fold cross-validation) we excluded it from the model and the selection process concluded.

We then externally validated the model on the hold-out period of data (January 2013 - July 2014) using a Pearson correlation coefficient as a metric of correlation between Google-predicted and observed pertussis incidence in California. To ensure the Google-prediction model was not detecting other respiratory infections which may have similar symptoms as pertussis we also determined the correlation between weekly Google-predicted influenza-like illness activity in California (obtained from the Google Flu Trends website for the period January 2013 - July 2014) and observed pertussis activity in California during the hold-out period^16^.

Finally, we looked for evidence of a delay between Google pertussis-related queries and pertussis case reporting to determine if the model could be used to predict epidemic trends several weeks in advance. We fit comparative models using a 1, 2, 3 and 4 week time lag introduced into the weekly observed pertussis trends, and compared the adjusted R^2^ for each of these models. All statistical analyses were performed by Stata version 13.1 (StataCorp, College Station, Texas, USA).

## Results

We identified 68 candidate terms searched by Californian Google users related to pertussis. The top ten terms are listed in Table 1, and ranked with respect to the magnitude of the correlation between their trends in weekly search frequency and weekly pertussis activity trends. The two terms “whooping cough California” (coefficient = 0.46, 95% CI = 0.34 - 0.58, p < 0.001) and “whooping cough adults” (coefficient = 0.33, 95% CI = 0.18 - 0.49, p < 0.001) were retained in a final linear model fit from the training data with an adjusted R^2^ of 0.69 (for the model containing both the terms “whooping cough California” and “whooping cough adults”) and a cross validation *r* = 0.83 (p<0.001). Model estimates were used to predict pertussis incidence during a second epidemic wave in the external hold-out set of data (Figure 1), with a Pearson correlation between predicted and actual pertussis incidence of 0.88 (p < 0.001, R^2^ = 0.77).

The peak predicted incidence was synchronous with the peak observed pertussis incidence although there was considerable underestimation of the epidemic wave magnitude (Figure 1). We further explored this underestimation of the epidemic magnitude by using adult-only pertussis cases to fit the model and determine its accuracy in the hold-out period. Due to the low proportion of pertussis cases diagnosed in adults (median 20%, IQR = 15 – 27%) we used weekly absolute case number rather than weekly incidence. Using this subset of data, the linear model with the terms “whooping cough California” and “whooping cough adults” had a worse fit (adjusted R^2^ = 0.66) than the same model using child cases alone (adjusted R^2^ = 0.69). The predicted weekly adult pertussis count trends in the hold-out period was more poorly correlated with observed weekly adult pertussis count trends (*r* = 0.77, p < 0.001) compared to the original model using child and adult data.

Pearson correlation between observed pertussis trends (Jan 2013 through July 2014) in California and Google Flu Trend estimates was very poor (*r* = -0.15), suggesting that the model was not predominantly detecting seasonal influenza-like illness which could be mistaken for pertussis.

Comparative extended models fitted with a 1,2, 3 and 4 week lag in the training period observed pertussis case incidence time series had an adjusted R^2^ of 0.65, 0.61, 0.57 and 0.53 respectively, possibly consistent with worsening prediction error with an increasing lag between time of Google search activity and pertussis reporting.

**Top ten candidate Google predictor search terms and their correlation coefficients:**
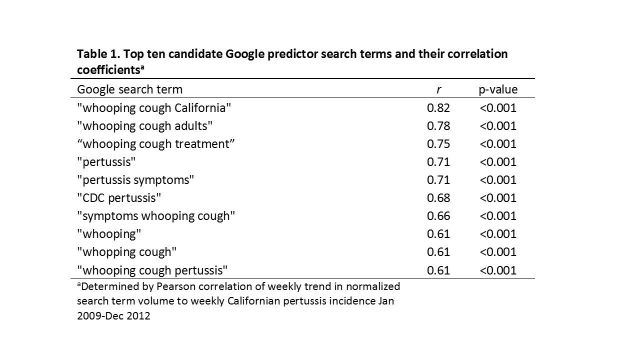

**Predicted and observed weekly pertussis incidence in California during the external hold-out period Jan 2013-July 2014.**
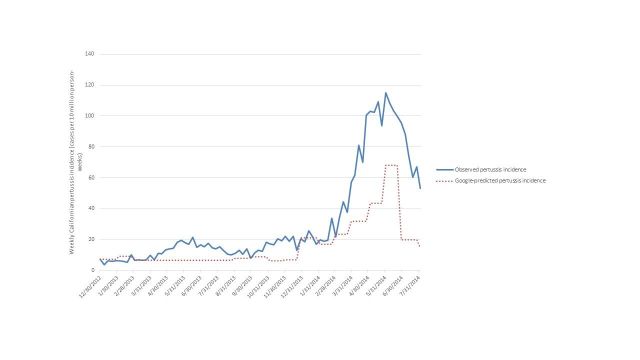

## Discussion

We were able to fit a moderately accurate predictive Californian pertussis Google model with precise ‘now-casting’ of peak pertussis incidence timing (that is, immediate estimation of the epidemic peak timing, which would be available before subsequent release of official aggregated data^17^), albeit it with substantial underestimation of peak incidence magnitude. We show some evidence that the model is specific for pertussis infections (rather than coincidentally detecting trends of other more respiratory viruses which may be confused with pertussis by patients or clinicians).

Our findings offer a free real-time surveillance signal that could be used, cautiously, with other data sources to augment public health monitoring of pertussis, particularly when conventional surveillance systems experience significant delays in reporting, consolidation or dissemination.

Based on linear model metrics such as *r* and R^2^, our regional model was more accurate than published national Google norovirus models (R^2^ = 0.74), and the worst documented performances of Google Dengue Trends (*r *= 0.82) and Google Flu Trends (R^2^ = 0.57). However both Google Flu and Dengue Trends have documented accuracy as high as *r* = 0.96 and *r* = 0.99 respectively^5^^,^ 6 ^,^ 8 ^,^ 18 and they generally outperformed our pertussis model. There are several possible reasons for this. Pertussis epidemics are generally smaller than influenza and dengue epidemics and this may have limited the model fitting. In addition, pertussis doesn’t have as regular seasonal epidemiology as influenza, and part of Google Flu Trend’s accuracy has been attributed to detecting search activity associated with winter rather than influenza *per se* 19. Additionally, Google returns trends of low volume search terms in monthly rather than weekly series, necessitating conversion from monthly to weekly trends with some likely misclassification. Changes to the availability in Google Trend data functions could thus perhaps improve the accuracy of Google pertussis predictive models for California and perhaps other regions within the United States. Finally, our model was fit and assessed on two epidemic waves four years apart, with enough of an interval so that there may have been changes in the public’s search behaviour of predictor terms selected in the first period of data.

The underestimation of predicted peak incidence magnitude was a particular limitation of this predictive model. This may be due to the large proportion of cases being undiagnosed in adults, which is suggested in literature and supported by our data which indicated that only around one fifth of pertussis cases during the study period were adults^20^. Indeed our sensitivity analysis indicated the model was less accurate when limited to adult data, even though one of model terms was specifically about adult pertussis cases (“whooping cough adults”).

We examined the possible role of a delay to pertussis diagnosis (from symptom onset) limiting the model performance, but an extended models using lagged observed pertussis time series did not show improvement of model fit. This may indicate that the model is detecting real-time Google activity performed around the time patients receive a pertussis diagnosis (rather than detecting Google activity about pertussis symptoms before seeking healthcare).

Since the launch of Google Flu Trends in 2009, there have been numerous other applications of Google Trends to a broad number of communicable diseases as diverse as Ebola and methicillin-resistant Staphylococcus aureus 18 ^,^ 21 ^,^ 22. It remains unclear, to our knowledge, whether any Google disease models are being implemented in actual public health practice or have ever triggered any public health response beyond that prompted by conventional surveillance. Concerns regarding the more recent performance of Google Flu Trends despite updated models emphasizes caution in the implementation of any Google-based epidemic prediction system^8^. The very recent decision by Google to cease public access to their Google Flu Trends and Google Dengue Trends predictions further underscores the need for further, robust study in this field^16^. Our findings help to further define the limitations and potential of Google-based epidemiological methods for enhanced communicable disease surveillance.

## Competing Interest

The authors have declared that no competing interests exist.
